# A Two-Stage Association Study Suggests BRAP as a Susceptibility Gene for Schizophrenia

**DOI:** 10.1371/journal.pone.0086037

**Published:** 2014-01-15

**Authors:** Fuquan Zhang, Chenxing Liu, Yong Xu, Guoyang Qi, Guozhen Yuan, Zaohuo Cheng, Jidong Wang, Guoqiang Wang, Zhiqiang Wang, Wei Zhu, Zhenhe Zhou, Xingfu Zhao, Lin Tian, Chunhui Jin, Janmin Yuan, Guofu Zhang, Yaguang Chen, Lifang Wang, Tianlan Lu, Hao Yan, Yanyan Ruan, Weihua Yue, Dai Zhang

**Affiliations:** 1 Wuxi Mental Health Center of Nanjing Medical University, Wuxi, Jiangsu Province, China; 2 Key Laboratory of Mental Health, Ministry of Health, Institute of Mental Health, The Sixth Hospital, Peking University, Beijing, China; 3 Department of Psychiatry, First Hospital of Shanxi Medical University, Taiyuan, China; 4 Peking-Tsinghua Center for Life Sciences, Beijing, China; 5 PKU-IDG/McGovern Institute for Brain Research, Peking University, Beijing, China; University of Illinois at Chicago, United States of America

## Abstract

Schizophrenia (SZ) is a neurodevelopmental disorder in which altered immune function typically plays an important role in mediating the effect of environmental insults and regulation of inflammation. The breast cancer suppressor protein associated protein (BRAP) is suggested to exert vital effects in neurodevelopment by modulating the mitogen-activated protein kinase cascade and inflammation signaling. To explore the possible role of BRAP in SZ, we conducted a two-stage study to examine the association of BRAP polymorphisms with SZ in the Han Chinese population. In stage one, we screened SNPs in BRAP from our GWAS data, which detected three associated SNPs, with rs3782886 being the most significant one (P  =  2.31E-6, OR  =  0.67). In stage two, we validated these three SNPs in an independently collected population including 1957 patients and 1509 controls, supporting the association of rs3782886 with SZ (P  =  1.43E-6, OR  =  0.73). Furthermore, cis-eQTL analysis indicates that rs3782886 genotypes are associated with mRNA levels of aldehyde dehydrogenase 2 family (ALDH2) (P  =  0.0039) and myosin regulatory light chain 2 (MYL2) (P < 1.0E-4). Our data suggest that the BRAP gene may confer vulnerability for SZ in Han Chinese population, adding further evidence for the involvement of developmental and/or neuroinflammatory cascades in the illness.

## Introduction

Schizophrenia (SZ) is a common yet disabling mental disorder characterized by profound disruption in cognition and emotion, affecting the most fundamental human attributes: language, thought, perception, affect, and sense of self. The onset of the illness is typically in late adolescence or early adulthood. Epidemiological research show that a variety of pre- and peri-natal insults are associated with increased vulnerability to SZ [1].

Since its initial proposition in late 1980’s [2], [3], the neurodevelopmental hypothesis has received much support from epidemiological, developmental and neuroimaging studies [4] and has been the dominant paradigm for schizophrenia research over the past two decades [5]. This hypothesis posits that SZ has its roots in disturbed development of the nervous system, in which cerebral insults occur during early brain development long before the full-blown of the illness. On the other hand, immune system may modulate normal neurodevelopment and there is increasing evidence for altered inflammatory factors in the etiology and pathophysiology of SZ [6]. For example, SZ has been shown to be associated with activated peripheral and central inflammatory responses; cytokines such as interleukin (IL)-1β, IL-6, and tumor necrosis factor α (TNF-α) participate in regulating normal brain development and have been implicated in abnormal corticogenesis [7].

The developmental formation of the mammalian central nervous system (CNS) requires precise spatial and temporal integration of mitogenic and neurogenic signals. The mitogen-activated protein kinase (MAPK) pathway plays a vital role in controlling cell proliferation and differentiation in the developing CNS, since the MARK pathway regulates various physiological signaling inputs and serves as a relay route from the cell surface to the nucleus [8]. The breast cancer suppressor protein (BRCA1) associated protein (BRAP) is a cytoplasmic protein which regulates nuclear targeting by retaining proteins with a nuclear localization signal in the cytoplasm [9]. BRAP, previously known as impedes mitogenic propagation (IMP), is a Ras effector and a negative regulator of the MAPK scaffold protein kinase suppressor of Ras 1 (KSR1) [10]. Thus, BRAP is regarded as a threshold modulator that controls the sensitivity of MAPK signaling, allowing adaptations, and maintains homeostasis in a complex tissue environment [11]. Such threshold regulation is specifically important in CNS development, during which each progenitor cell has a unique environment and its fate choices are precisely correlated to its spatial location. In addition, BRAP is proposed to be a modulator that associates with Skp1-Cullin1-F-box protein (SCF) complex and controls TNF-a-induced nuclear factor-kappaB (NF-κB) nuclear translocation; while NF-κB is critical for the expression of multiple genes involved in inflammatory responses and cellular survival [12]. BRAP is also an anchor protein for p21 through direct interaction [13]. These lines of evidence suggest that BRAP can control different kinds of intracellular signals. Interestingly, recent genetic analyses have revealed that BRAP is associated with several human disorders caused by inflammatory dysfunction, including myocardial infarction, carotid atherosclerosis and central obesity [14]-[16].

Given the close link between developmental neuroinflammation and SZ, we hypothesized that the BRAP gene may influence the genesis of SZ. In this work, we aimed to evaluate the association of BRAP polymorphisms with SZ in a two-stage study. In the discovery stage, we derived relevant data from our GWAS data [17], involving six SNPs in 768 SZ cases and 1348 healthy controls. In the validation stage, we replicated the most significant SNPs in 1957 cases and 1509 controls. Finally, we sought to explore the potential role the associated SNP(s) using cis-eQTL (expression quantitative trait loci) analysis via online datasets.

## Materials and Methods

### Subjects

All participants were unrelated Han Chinese recruited from the North of China. The GWAS sample consisted of 768 unrelated subjects with SZ (360 males and 408 females) and 1348 control subjects (658 males and 690 females). For validation, an independent sample consisting of 1957 cases (1037 males and 920 females) and 1509 controls (360 males and 1149 females) was recruited from northern China. The consensus diagnoses were made by at least two experienced psychiatrists according to the Diagnosis and Statistical Manual of Mental Disorders Fourth Edition (DSM-IV) criteria for schizophrenia. None of the patients had severe medical complications. Healthy controls were recruited from communities with simple non-structured interview performed by psychiatrists, who excluded individuals with history of mental health and neurological diseases.

The study was approved by the Medical Research Ethics Committee of the Institute of Mental Health, Peking University. All participants enrolled in the study signed written informed consent.

### Genotyping

Peripheral blood samples were collected from all subjects. Genomic DNA was extracted using the Qiagen QIAamp DNA Mini Kit. In the screening stage, we derived genotypes of six SNPs in BRAP from our GWAS data [17], involving rs1544396, rs11065987, rs10744774, rs17628828, rs3782886, and rs601663. In the validation stage, the genotypes of rs1544396, rs3782886, and rs601663 were determined using the Sequenom MassARRAY system (Sequenom iPLEX). 1957 cases and 1059 controls were successfully genotyped, the calling rate being 99.91%. Data from this study are freely available upon request.

### Bioinformatics Analyses

Genetic association tests were analyzed using PLINK 1.07 [18], and 10000 permutations were used to achieve the adjusted P values. Linkage disequilibrium (LD) among the markers was plotted with Haploview [19]. The pie chart of the worldwide distribution of rs3782886 was created from HGDP Selection Browser [20]. Genetic power was estimated by PS [21]. We performed the cis-eQTL using Genevar 3.2 [22] in two Asian populations from HapMap3 project: CHB (80 Han Chinese from Beijing, China) and JPT (82 Japanese in Tokyo, Japan) [23], which analyzed the association of rs3782886 genotypes with neighboring genes within 1 Mb distance. Protein-protein interaction network was built via STRING v9.1 [24]. Finally, we predicted the function of rs3782886 via the on-line tool F-SNP (http://compbio.cs.queensu.ca/F-SNP/) [25].

## Results

### Genetic association

**Figure 1:**
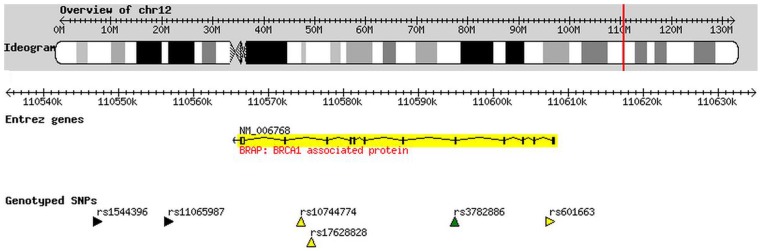
Genomic location of the BRAP gene and the six SNPs.

**Figure 2:**
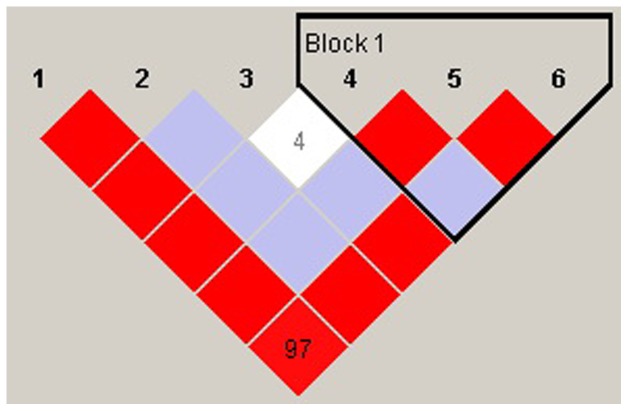
LD plot among the SNPs.

**Figure 3:**
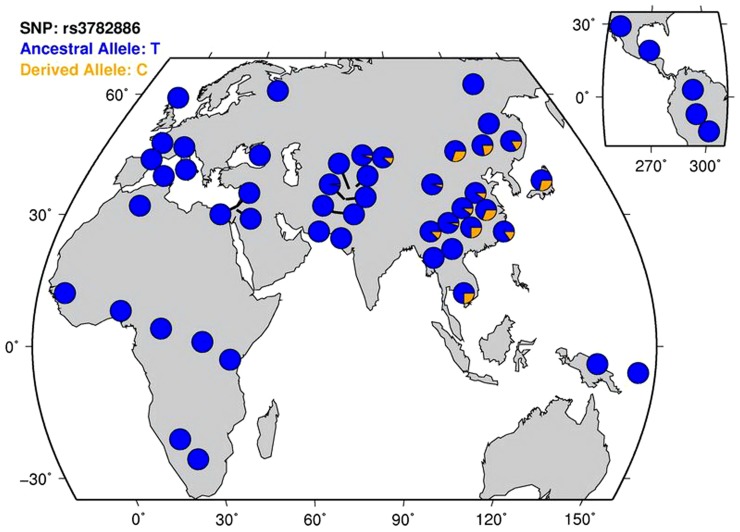
Worldwide allele frequency distribution of rs3782886.

Genotypic distributions of the six SNPs did not deviate from Hardy-Weinberg equilibrium (HWE) in either the patient group or the control group (P > 0.05). The BRAP maps on chromosome 12q24, and the six SNPs span across the gene (Figure 1). The LD plot among the SNPs is shown in Figure 2. Three SNPs were found to be associated with SZ, including rs1544396 (P  =  2.75E-5, adjusted P  =  2.00E-4, OR  =  1.63), rs3782886 (P  =  2.31E-6, adjusted P  =  1.00E-4, OR  =  0.67), and rs601663 (P  =  0.0047, adjusted P  =  0.025, OR  =  1.56) (Table 1). The most significant SNP, rs3782886, was replicated in the validation data (P  =  1.43E-6, adjusted P  =  1.00E-4, OR  =  0.73), while the other two SNPs failed to be replicated. Thus, rs3782886 was considered be consistently associated with SZ across the two samples. The allele frequencies of rs3782886 across the world is shown in Figure 3.

**Table 1:** Allelic distributions of BRAP polymorphisms in patients and controls.

Stage	SNP	1/2	SZ (MAF)	Control (MAF)	OR (95% CI)	P	P__perm_
1	rs1544396	A/G	150/1386(0.098)	168/2528(0.062)	1.63(1.29–2.05)	2.75E-5	2.00E-4
	rs11065987	G/A	8/1528(0.005)	7/2689(0.003)	2.01(0.73–5.56)	0.169	0.630
	rs10744774	C/A	7/1529(0.005)	7/2687(0.003)	1.76(0.62–5.02)	0.286	0.868
	rs17628828	A/G	24/1512(0.016)	29/2667(0.011)	1.46(0.85–2.52)	0.171	0.649
	rs3782886	G/A	237/1299(0.154)	576/2118(0.214)	0.67(0.57–0.79)	2.31E-6	1.00E-4
	rs601663	A/G	77/1459(0.05)	88/2608(0.033)	1.56(1.14–2.14)	0.0047	0.025
2	rs1544396	A/G	337/3571(0.086)	254/2764(0.084)	1.03(1.02–1.03)	0.76	0.984
	rs3782886	G/A	534/3374(0.137)	540/2478(0.179)	0.73(0.72–0.73)	1.43E-6	1.00E-4
	rs601663	A/G	156/3752(0.04)	133/2885(0.044)	0.9(0.9–0.91)	0.392	0.733

MAF: minor allele frequency; P__perm_: adjusted P of 10000 permutations.

### cis-eQTL analysis and SNP function prediction

**Figure 4:**
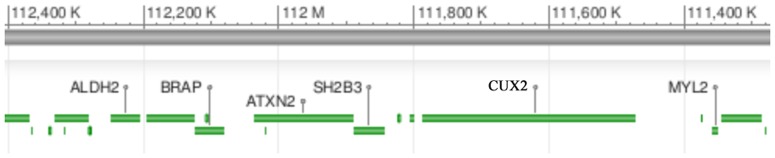
Neighboring genes of BRAP.

**Figure 5:**
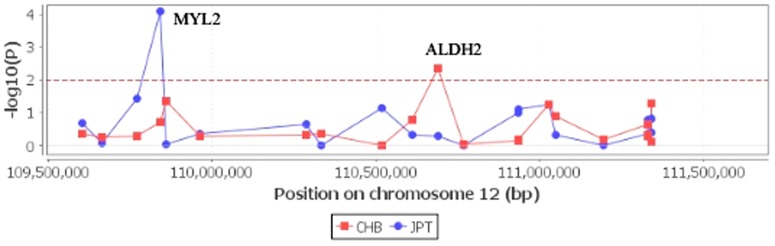
cis-eQTL analysis. Two genes, ALDH2 and MYL2, were associated with rs3782886 genotypes in the Chinese (CHB) and Japanese (JPT) populations, respectively.

**Figure 6:**
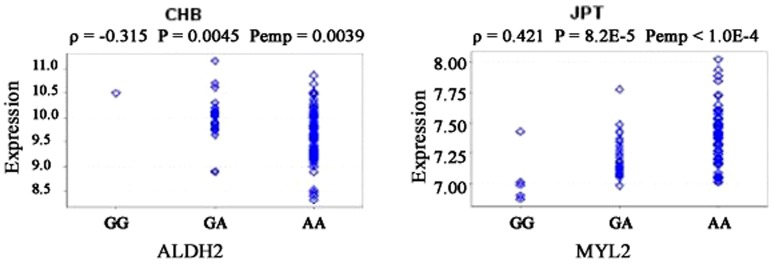
Correlation of rs3782886 genotypes with mRNA levels of ALDH2 (left) and MYL2 (right). ρ: Spearman's rank correlation coefficient. Pemp: Adjusted P-value from 10000 permutation tests.

**Figure 7:**
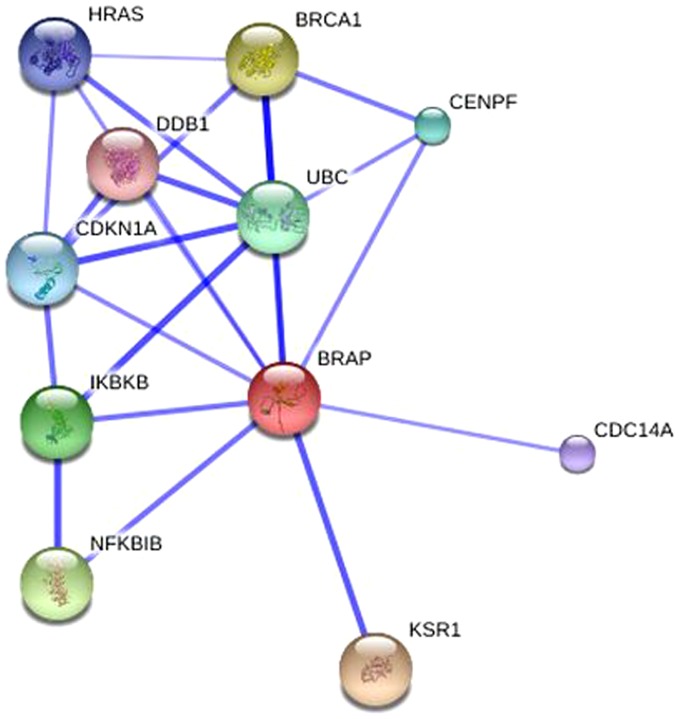
Protein interaction network of BRAP.

Several genes are located near the BRAP gene on chromosome 12q24, including aldehyde dehydrogenase 2 family (ALDH2) and myosin regulatory light chain 2 (MYL2) (Figure 4). Of note is that BRAP is located at the upper stream of both ALDH2 and MYL2. The cis-eQTL analysis detected two associated genes (Figure 5). There is an inverse correlation between the dosage of rs3782886 A-allele and ALDH2 mRNA levels in the Han Chinese population (P_permutation_  =  0.0039), with the Spearman's rank correlation coefficient (ρ) being –0.315; while, the dosage of rs3782886 A-allele correlates positively with the MYL2 mRNA levels in the Japanese population (P_permutation_ < 1.0E-4, ρ  =  0.421, Figure 6). SNP rs3782886 was predicted to influence several transcriptional factors, and its functional significance (FS) score is 0.5, suggesting a possible role of in gene function (the median FS score for neutral SNPs is 0.176, whereas, for disease-related SNPs, the median rises to 0.5, Figure S1) [26]. rs3782886 is predicted to interact directly with several other proteins (Figure 7).

## Discussion

SZ is one of most disabling psychoses, affecting virtually all brain functions. Although the cause of this disorder remains elusive, by far the vast majority of evidence points to the neurodevelopmental model in which abnormalities of early brain development predispose to future onset of SZ. And it has been postulated that immune system exerts an effect on normal CNS development while abnormal immune reaction may adversely impede this process. Empirical evidence indicates that immune-mediated disruption of early brain development contributes to the precipitation of later psychotic disease [6]. Salient examples include roles of viral infection [27] and oxidative stress [28] in the genesis of SZ. Thus, both neuroprogression and neuroinflammation process could exert pivotal roles in human CNS maturation and functioning.

SZ is a complex genetic disease with an estimated heritability of approximately 80–85% [29]. Genetic epidemiology and population genetics suggest that a spectrum of genetic risk variants from a host of candidate genes contribute to the disorder. Mounting work has revealed lots of insight into the genetic underpinnings of SZ, nevertheless, the larger bulk of disease heritability remains unaccounted for [30]. Part of the problem may lie in the fact that most efforts have been focusing on the dopamine system in the search of its genetic basis, with little emphasis on neurodevelopmental pathway genes.

BRAP is a polytrophic molecule which plays vital roles in several signaling pathways. First, it is a threshold regulator in the MAPK pathway, while the MAPK cascade forms the backbone of cell signaling and has an extraordinarily broad impact in cell growth and differentiation. Second, BRAP also acts as a key mediator of inflammatory cascades by regulating NF-κB nuclear translocation [15], while the immune system participates in the neural progression. Thus, BRAP acts more as a molecular buffer to maintain homeostasis of a cell’s response to the environment [11], and is critical to cerebral development as well as brain function. Apart from KSR1 and BRCA1, protein-protein network analysis suggests several other interacting partners. Of note is ubiquitin C (UBC), a critical ‘switchboard’ node which was implicated in SZ by a mRNA expression study [31].

BRAP is located on chromosome 12q24, one of the SZ-related chromosomal fragile sites [32] harboring several candidate genes for SZ, including nitric oxide synthase 1 (NOS1) [33] and D-amino acid oxidase (DAO) [34]. SNP rs11066001 lies in intron 3, while rs3782886 is a synonymous SNP in exon 5. Of note is that rs11066001 is in LD with rs3782886, with R^2^ of 0.891 and D` of 1 in our data. Some studies reported that two SNPs—rs11066001 and rs3782886—in BRAP were significantly associated with coronary artery disease in Asian populations [14], [35], and metabolic syndrome in the Han Chinese population [36]. Another study reported the association of rs11066001 with carotid atherosclerosis in Taiwanese [15]. Notably, all these studies were conducted in Asian populations. According to HapMap data, these two SNPs are nonpolymorphic in European (CEU) and African (YRI) population, therefore rs3782886 seems likely to be present chiefly in Asian populations (Figure 3). However, another SNP (rs11065987) in BRAP was reported to be associated with metabolic syndrome in European Americans and African Americans [16], suggesting genetic heterogeneity of BRAP across diverse populations.

In this work, we genotyped and analyzed six polymorphisms within BRAP, detecting three significant SNPs: rs1544396, rs3782886, and rs601663. The most significant SNP, rs3782886, was replicated in the validation data, while the other two SNPs failed to be replicated. Thus, our data strongly support the association of rs3782886 with SZ in Han Chinese population, with the mutant G-allele conferring a protective effect against SZ disease (OR  =  0.68).

The cis-eQTL analysis revealed that genotypes of rs3782886 were associated with mRNA levels of two nearby genes—ALDH2 and MYL2—in the Chinese and Japanese population, respectively. rs3782886 was predicted to influence several transcriptional factors [25]. It is noteworthy that rs3782886 is located at the upper stream of both ALDH2 and MYL2. Considering that the upper stream of a gene harbors vital regulating elements for transcription, it is plausible that such a cis-regulating effect may exist.

The ALDH2 protein is the mitochondrial isoform of aldehyde dehydrogenase, which metabolizes acetaldehyde and governs the detoxification of acetaldehyde formed following alcohol consumption and the ultimate elimination of alcohol from the body [37]. In addition to metabolizing acetaldehyde, aldehyde dehydrogenase is also involved in dopamine and serotonin metabolism [37]. Genetic studies suggested that ALDH2 was associated with alcohol dependence [38], bipolar I disorder [39], and cardiovascular diseases [37]. Interestingly, both BRAP and ALDH2 were found to be associated with coronary artery disease in the Japanese [40].

MYL2 encodes the myosin regulatory light chain that specifically binds to myosin heavy chains, constituting one of the most important components of sarcomere filaments [41]. MYL2 also mediates the AMP-activated protein kinase (AMPK) pathway which regulates energy homeostasis in eukaryotes [42]. This gene was found to be linked with multiple cardiovascular diseases [43] and drinking behavior [44].

Considering the involvement of dopamine and serotonin in SZ and the comorbidity of SZ with alcohol dependence and cardiovascular diseases, it is plausible that these two genes (ALDH2 and MYL2) may be related to SZ. The relationship of rs3782886 genotypes with BRAP mRNA is unknown because of lacking the BRAP data in the dataset [22], nevertheless, a link between them may possibly exist since rs3782886 can affect the transcriptional activity of the gene [14]. Together, these data suggest that rs3782886 may represent effects beyond the BRAP gene, possible through direct cis-regulation or through proxy effect by in LD with the truly regulatory SNPs.

Assuming a disease allele frequency of 0.20, and a type I error of 0.05, our sample has a 93.8% power to detect a variant with an odds ratios of 0.73 [21]. The major limitation may be the selection of SNPs were not unbiased, but relied on the design of the commercial array. Due to constraint of fund, we were not able to biologically validate the role of rs3782886 on the gene function, which is warranted to be explored in the future.

In conclusion, our findings suggest BRAP as a candidate gene for SZ the Han Chinese population. Our data provide novel genetic evidence for the involvement of developmental and/or neuroinflammatory cascades in SZ.

## Supporting Information

Figure S1 **Predicted functions of rs3782886 (** http://compbio.cs.queensu.ca/F-SNP/ **). FS: functional significance.**
(TIF)
