# Supplementary figures and images for: A Two-Stage Association Study Suggests BRAP as a Susceptibility Gene for Schizophrenia
Source: PLoS One. 2014 Jan 15;9(1):e86037. doi: 10.1371/journal.pone.0086037 (PMC3893271; doi:10.1371/journal.pone.0086037)

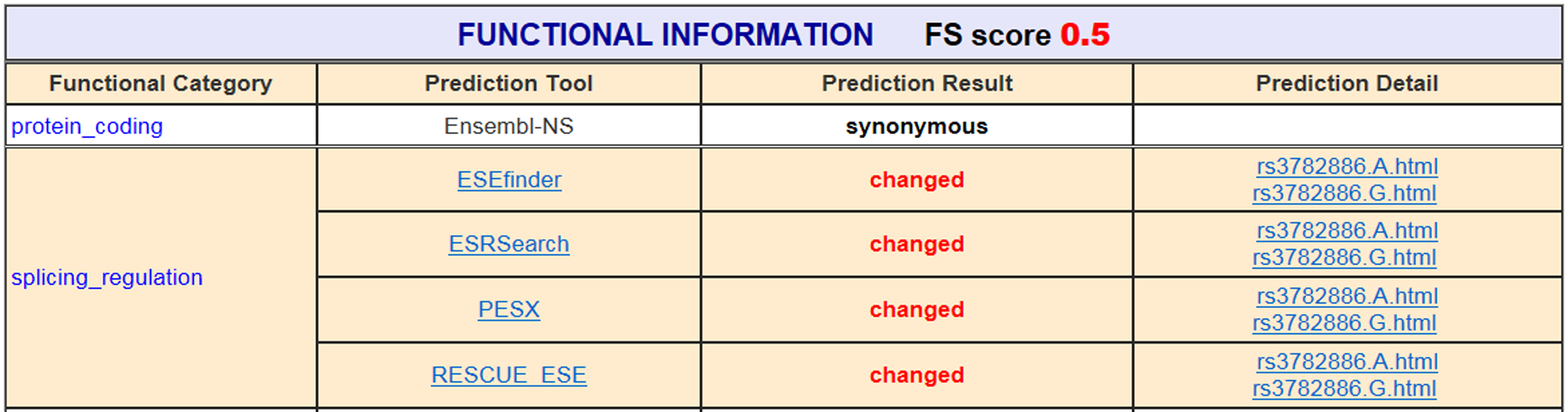

Supplement: Figure S1 — Predicted functions of rs3782886 ( http://compbio.cs.queensu.ca/F-SNP/ ). FS: functional significance. (TIF) [file pone.0086037.s001.tif]
